# A martius flap in the treatment of iatrogenic distal urogenital fistula

**DOI:** 10.1590/S1677-5538.IBJU.2017.0444

**Published:** 2018

**Authors:** Ivan Ignjatovic, Dragoslav Basic, Milan Potic, Ljubomir Dinic, Aleksandar Skakic

**Affiliations:** 1 Clinical Center Nis, Serbia; 2 University in Nis, Faculty of Medicine Nis, Serbia

## Abstract

**Introduction:**

Distal urogenital fistulas (DUF) are usually iatrogenic and are uncommon in Europe. They occur in the urethra or near the bladder neck, and can be caused by vaginal hysterectomy, para-urethral cyst surgery, or erosion of the bladder or urethra from tension-free slings or meshes. The psychological and physical health consequences of DUF are devastating because most patients consider themselves “healthy” before surgery. Incontinence can appear after successful DUF closure due to previously occult incontinence or urethral incompetence. Additional surgery for incontinence is sometimes necessary to achieve satisfactory outcome.

**Materials and Methods:**

A Martius flap was used in 23 patients between 2000 and 2015. Patient age range was 38-75 years (mean, 58.7). DUF was due to gynecologic surgery for benign disease (15 / 23; 65.2%), mesh / sling erosion (2 / 23; 8.7%), and malignancy (6 / 23; 26.1%). The follow-up period was one year.

**Results:**

DUF was closed in 22 patients (95.6%). Satisfaction and complete dryness was achieved in 16 patients (69.6%) after the first procedure. Postoperative complications were: postoperative hematoma in 1 (4.4%), primary failure in 1 (4.4%), overactive bladder (OAB) syndrome in 3 (13.2%) and postoperative incontinence in 6 (26.4%) patients. A fascial sling was placed in patients with incontinence. All patients were dry after the secondary surgery. Anticholinergics were used for the treatment of OAB syndrome. Discomfort at the flap harvesting site was of minor importance. Finally, 22 out of 23 patients (95.6%) were satisfied.

**Conclusion:**

A Martius flap and additional fascial sling could be successfully used to optimize DUF treatment.

## ARTICLE INFO

**Available at: http://www.intbrazjurol.com.br/video-section/20170444_Ignjatovic_et_al**

**Int Braz J Urol. 2018; 44 (Video #21): 1265-5**

